# Concentration Dependent Effect of Plant Root Exudates on the Chemosensory Systems of *Pseudomonas putida* KT2440

**DOI:** 10.3389/fmicb.2019.00078

**Published:** 2019-01-30

**Authors:** Diana López-Farfán, José A. Reyes-Darias, Miguel A. Matilla, Tino Krell

**Affiliations:** Estación Experimental del Zaidín, Department of Environmental Protection, Consejo Superior de Investigaciones Científicas, Granada, Spain

**Keywords:** chemotaxis, chemoreceptor, *Pseudomonas*, root exudates, root colonization

## Abstract

Plant root colonization by rhizobacteria can protect plants against pathogens and promote plant growth, and chemotaxis to root exudates was shown to be an essential prerequisite for efficient root colonization. Since many chemoattractants control the transcript levels of their cognate chemoreceptor genes, we have studied here the transcript levels of the 27 *Pseudomonas putida* KT2440 chemoreceptor genes in the presence of different maize root exudate (MRE) concentrations. Transcript levels were increased for 10 chemoreceptor genes at low MRE concentrations, whereas almost all receptor genes showed lower transcript levels at high MRE concentrations. The exposure of KT2440 to different MRE concentrations did not alter c-di-GMP levels, indicating that changes in chemoreceptor transcripts are not mediated by this second messenger. Data suggest that rhizosphere colonization unfolds in a temporal fashion. Whereas at a distance to the root, exudates enhance chemoreceptor gene transcript levels promoting in turn chemotaxis, this process is reversed in root vicinity, where the necessity of chemotaxis toward the root may be less important. Insight into KT2440 signaling processes were obtained by analyzing mutants defective in the three *cheA* paralogous genes. Whereas a mutant in *cheA1* showed reduced c-di-GMP levels and impaired biofilm formation, a *cheA2* mutant was entirely deficient in MRE chemotaxis, indicating the existence of homologs of the *P. aeruginosa* *wsp* and *che* (chemotaxis) pathways. Signaling through both pathways was important for efficient maize root colonization. Future studies will show whether the MRE concentration dependent effect on chemoreceptor gene transcript levels is a feature shared by other species.

## Introduction

Bacteria adapt to changing environmental conditions through different signal transduction systems that most commonly include one- and two-component systems as well as chemosensory pathways (Galperin, 2005; Laub and Goulian, 2007; Hazelbauer et al., 2008). In the latter systems, the direct binding of chemoeffectors or chemoeffector-loaded periplasmic binding proteins to the ligand binding domain (LBD) of chemoreceptors causes a molecular stimulus that is transmitted across the membrane where it modulates the autokinase activity of the CheA histidine kinase and consequently transphosphorylation to the CheY response regulator, which generates ultimately the pathway output (Hazelbauer et al., 2008). Typically, the adaptation of pathway sensitivity to the ambient chemoeffector concentration is achieved through the action of CheR methyltransferases and CheB methylesterases. Chemosensory pathways have been initially described to mediate chemotaxis (Parkinson et al., 2015). However, other studies show that these pathways can also be responsible for type IV pili-based motility (Whitchurch et al., 2004) or mediate alternative cellular functions (Wuichet and Zhulin, 2010).

*Escherichia coli* is the traditional model to study chemosensory pathways. This bacterium has four chemotaxis and an aerotaxis receptor that feed into a single pathway (Parkinson et al., 2015). However, the complexity of chemosensory signaling mechanisms depends on the bacterial lifestyles (Alexandre et al., 2004; Lacal et al., 2010b). Metabolically versatile bacteria that are able to inhabit different ecological niches were found to possess many more chemoreceptors, up to 80, whereas niche-specific bacteria contain fewer receptors (Lacal et al., 2010b). In addition, many bacteria possess multiple chemosensory pathways (Hamer et al., 2010; Wuichet and Zhulin, 2010), which adds to the complexity.

The colonization of plant roots by beneficial rhizobacteria is a process of enormous relevance since it was shown promote plant growth and to induce a systemic resistance of plants to different pathogens (Pieterse et al., 2014; Vejan et al., 2016). Chemotaxis of beneficial bacteria to plant root exudates is a necessary requisite for efficient root colonization (de Weert et al., 2002; Allard-Massicotte et al., 2016; Scharf et al., 2016). Plant root exudates are very complex compound mixtures of primary and secondary metabolites (Badri and Vivanco, 2009; van Dam and Bouwmeester, 2016; Petriacq et al., 2017) and chemotaxis to many individual constituents of root exudates, mainly primary metabolites, has been reported (Webb et al., 2016; Feng et al., 2018; Matilla and Krell, 2018). In this study we have used maize root exudates (MRE) that were shown in previous studies to contain primary metabolites like sugars, sugar acids, organic acids and amino acids (Kraffczyk et al., 1984; Carvalhais et al., 2011; Fan et al., 2012). In addition, a large series of other compounds were detected in MRE such as benzylamines, polyamines, pyrrol derivatives, fatty acids, nucleotide derivatives, purines, steroids, phytohormones, phenylpropanoids, terpenoids, flavonoids, and benzoxazinoids (Kidd et al., 2001; Neal et al., 2012; da Silva Lima et al., 2014; Petriacq et al., 2017). Although these latter compounds are generally exuded in smaller quantities, some of them were shown to be important in plant-to-microorganism signaling (Hassan and Mathesius, 2012).

*Pseudomonas putida* strains are metabolically versatile and respond chemotactically to a wide range of compounds (Parales et al., 2015; Sampedro et al., 2015). To analyze the influence of MRE on chemoreceptor gene transcript levels, we have used *P. putida* KT2440 (Belda et al., 2016) in this study as a model organism. This strain was isolated from soil, is nutritionally versatile, has a saprophytic lifestyle, colonizes plant roots efficiently and is able to protect plants against phytopathogens through the induction of systemic resistance or using type VI secretion systems (Bagdasarian et al., 1981; Espinosa-Urgel et al., 2002; Nakazawa, 2002; Regenhardt et al., 2002; Matilla et al., 2010; Bernal et al., 2017). KT2440 has 27 different chemoreceptors and research over mainly the last decade has permitted to gain insight into the function of more than half of them (Table 1). These chemoreceptors were found to respond to different organic and amino acids, polyamines, purine bases, gamma-aminobutyric acid, cyclic organic acids, inorganic phosphate or changes in the energy status.

**Table 1:** *Pseudomonas putida* KT2440 chemoreceptors.

Locus tag (chemoreceptor name)	Chemoeffector/comment	Reference
PP_0317 (McpR)	Succinate, malate, fumarate	Parales et al., 2013
PP_0320 (McpH)	Adenine, guanine, hypoxanthine, xanthine, uric acid, purine	Fernandez et al., 2016
PP_0562 (CtpL_PP)	Inorganic phosphate (by homology with *P. aeruginosa*)	Wu et al., 2000; Ortega et al., 2017
PP_1228 (McpU)	Putrescine, spermidine, cadaverine, agmatine, ethylenediamine, histamine	Corral-Lugo et al., 2016; Corral-Lugo et al., 2018; Gavira et al., 2018
PP_1371 (McpG)	Gamma-aminobutyric acid (GABA)	Reyes-Darias et al., 2015
PP_1488 (WspA_PP)	Unknown/WspA homolog, mutation reduces biofilm formation	Corral-Lugo et al., 2016; Ortega et al., 2017
PP_2111 (Aer2)	Energy taxis	Sarand et al., 2008
PP_2120 (CtpH_PP)	Inorganic phosphate (by homology with *P. aeruginosa*)	Wu et al., 2000; Ortega et al., 2017
PP_2249 (McpA)	Gly, L-isomers of Ala, Cys, Ser, Asn, Gln, Phe, Tyr, Val, Ile, Met, Arg	Corral-Lugo et al., 2016
PP_2257 (Aer1)	Energy taxis?	Sarand et al., 2008
PP_2310	Mutation increases biofilm formation	Corral-Lugo et al., 2016
PP_2643 (PcaY_PP)	Different cyclic acids	Luu et al., 2015; Fernandez et al., 2017
PP_2861 (McpP)	Pyruvate, L-lactate, propionate, acetate	Garcia et al., 2015
PP_4521 (Aer3)	Energy taxis?	Sarand et al., 2008
PP_4658 (McpS)	Malate, fumarate, oxaloacetate, succinate, citrate, isocitrate, butyrate	Lacal et al., 2010a; Lacal et al., 2011; Pineda-Molina et al., 2012
PP_4888	Expression regulated by benzoxazinoids	Neal et al., 2012
PP_5020 (McpQ)	Citrate, citrate/metal^2+^	Martin-Mora et al., 2016


*This strain has 27 chemoreceptors and listed are the receptors for which information on their function are available. Indicated are the corresponding chemoreceptor ligands and references.*

KT2440 also has three copies of each of the chemosensory signaling proteins suggesting that they assemble to three different signaling pathways. Of the three *cheA* paralogs, *cheA1* (Supplementary Figure S1) is within a gene cluster that shares high sequence similarity with the *wsp* cluster of *P. aeruginosa* encoding a pathway that is not related to chemotaxis but that modulates c-di-GMP levels and consequently biofilm formation (Hickman et al., 2005). The notion that this pathway in KT2440 has a similar function is supported by the observation that the deletion of the *cheR1* gene of the same cluster largely reduced biofilm formation (Garcia-Fontana et al., 2013). Previous studies have shown that the deletion of *cheR2* (Supplementary Figure S1) abolished chemotaxis (Garcia-Fontana et al., 2013) and we hypothesize that *cheR2* and genes of the cluster harboring *cheA2* encode the proteins of a chemotaxis pathway. The function of *cheA3* (Supplementary Figure S1) and its neighboring signaling genes remains unknown.

We have published recently a study in which we have investigated the effect of different stimuli on the KT2440 chemoreceptor gene transcript levels (Lopez-Farfan et al., 2017). Apart from the culture medium and growth phase, we were able to show that many chemoeffectors regulate the transcript levels of their cognate chemoreceptor genes. In particular, different C2- and C3-carboxylic acids, amino acids, cyclic carboxylic acids, putrescine as well as different purine derivatives increased the transcript levels of their cognate chemoreceptor genes, whereas inorganic phosphate reduced transcript levels of both of its cognate receptor genes. Interestingly, most of these chemoeffectors were shown to be present in MRE (Carvalhais et al., 2011; Fan et al., 2012). In this study we investigate the effect of different MRE concentrations on chemoreceptor gene transcript levels. We show that responses depend largely on the MRE concentration, with increase in transcript levels at low MRE concentrations and a general reduction at elevated concentrations. Data reported provide novel insight into the nature and mechanisms of bacterial responses in the rhizosphere.

## Materials and Methods

### Strains, Plasmids and Primers

Bacterial strains and plasmids used in this study are listed in Table 2, whereas oligonucleotides are provided in Table 3.

**Table 2:** Strains and plasmids used in this study.

Strain or plasmid	Relevant characteristics^a^	Source
**Strains**		
*Escherichia coli*		
DH5α	*supE44 lacU169* (*Ø80lacZ*ΔM15) *hsdR17* (*r_k_*^-^*m_k_*^-^), *recA1 endA1 gyrA96 thi-1 relA1*	Woodcock et al., 1989
CC118λ*pir*	*araD* Δ(*ara, leu*) Δ*lacZ*74 *phoA*20 *galK thi-1rspE rpoB argE recA1* λ*pir*	Herrero et al., 1990
HB101	F^-^ Δ(*gpt-proA*)62 *leuB6 supE44 ara-14 galK2 lacY1* Δ(*mcrC-mrr*) *rpsL20* (Sm^R^) *xyl-5 mtl-1 recA13 thi-1*	Boyer and Roulland-Dussoix, 1969
BL21 (DE3)	F^-^ *ompT* *gal* *dcm* *lon* *hsdS_B_* (*r_B_*^-^*m_B_*^-^) λ(DE3)	Jeong et al., 2009
*Pseudomonas putida*		
KT2440	Wild type; Derivative of *P. putida* mt-2, cured of pWWO	Belda et al., 2016
KT2440R	Rif^R^ derivative of *P. putida* KT2440; wild type	Espinosa-Urgel and Ramos, 2004
KT2440RTn*7*-Sm	Extragenic site-specific insertion of miniTn*7*; Rif^R^, Sm^R^	Matilla et al., 2007
KT2440R_CheA1	*pp1492*::mini-tn*5*-Km; Rif^R^, Km^R^	Duque et al., 2007
KT2440R_CheA2	Δ*pp4338*::km; constructed by marker exchange, Rif^R^,Km^R^	This study
KT2440R_CheA3	*pp4988*::mini-tn*5*-Km; Rif^R^, Km^R^	Duque et al., 2007
		
**Plasmids**		
pET28b(+)	Km^R^; Protein expression plasmid	Novagen
pRK600	Cm^R^; *mob tra*	Finan et al., 1986
pUC18Not	Ap^R^; identical to pUC18 but with two NotI sites flanking pUC18 polylinker	Herrero et al., 1990
p34S-Km3	Km^R^, Ap^R^; Km3 antibiotic cassette	Dennis and Zylstra, 1998
pKNG101	Sm^R^; *oriR6K mob sacBR*	Kaniga et al., 1991
pCdrA::*gfp*^C^	Ap^R^, Gm^R^; FleQ dependent c-di-GMP biosensor	Rybtke et al., 2012
pBBR1MCS-5	Gm^R^; *oriRK2 mobRK2*	Kovach et al., 1995
pPP4888	Km^R^; pET28b(+) derivative containing DNA fragment encoding PP4888-LBD cloned into NdeI and BamHI sites	This study
pMAMV287	Ap^R^; 2.2-kb BamHI/HindIII PCR product containing a fragment of *pp4338* was inserted into the same sites of pUC18Not	This study
pMAMV289	Ap^R^, Km^R^; a 0.8-kb PstI fragment internal to *pp4338* of pMAMV287 was replaced for a 1-kb PstI fragment containing *km3* cassette of p34S-Km3	This study
pMAMV290	Sm^R^, Km^R^; 2.4-kb NotI fragment of pMAMV289 was cloned at the same site in pKNG101	This study


*^*a*^Ap, ampicillin; Gm, gentamycin; Km, kanamycin; Rif, rifampin; Sm, streptomycin*.

**Table 3:** Oligonucleotides used in this study.

Name	Sequence (5′–3′)	Description/purpose
PP4338-BamHI-F	TAATGGATCCAAGCGCGTCACCACACTG	Forward primer for mutagenesis of *PP_4338*
PP4338-HindIII-R	TAATAAGCTTGTACGCGACAGGTCGAGGTG	Reverse primer for mutagenesis of *PP_4338*
PP4888LBD-NdeI-F	AACATATGACCCGAAGCACGGTCACCGCC	Forward primer to clone the region encoding PP4888-LBD into pET28b(+)
PP4888LBD-BamHI-R	AAGGATCCCTAGCGCAGCTCGGCAGCCGCAT	Reverse primer to clone the region encoding PP4888-LBD into pET28b(+)
qPP_McpR_F	CATACTGGTTGGCGGCTTTT	RT-qPCR of *PP_0317* (*mcpR*)
qPP_McpR_R	TCCGAGCAGATCAACAAGGT	RT-qPCR of *PP_0317* (*mcpR*)
qPP_0320_F	ACCGGCCATTACTACAACGA	RT-qPCR of *PP_0320* (*mcpH*)
qPP_0320_R	ATGTTGATGAACCGTTCGGC	RT-qPCR of *PP_0320* (*mcpH*)
qPP_CtpL_F	TGCTGGTGACCGTGTGTATC	RT-qPCR of *PP_0562* (*ctpL_PP*)
qPP_CtpL_R	CCCAGATAGCGCTCCATCAG	RT-qPCR of *PP_0562* (*ctpL_PP*)
qPP_McpU_F	ATTGGCCTGTACCTGGTGTT	RT-qPCR of *PP_1228* (*mcpU*)
qPP_McpU_R	GGGACCAGTACAGCGAGAAA	RT-qPCR of *PP_1228* (*mcpU*)
qPP_McpG_F	CTTCCTCACGGTCTACCTGG	RT-qPCR of *PP_1371* (*mcpG*)
qPP_McpG_R	GTTCATGCCGTCCTTGTACC	RT-qPCR of *PP_1371* (*mcpG*)
qPP_1488_F	TGCTGATCAGGCATCCAGTC	RT-qPCR of *PP_1488* (*wspA_PP*)
qPP_1488_R	AAGCTTGGCATTGACCAGGT	RT-qPCR of *PP_1488* (*wspA_PP*)
qPP_2111_F	GAGATCAGCCGCAACATCAG	RT-qPCR of *PP_2111* (*aer2*)
qPP_2111_R	GTCAGTTCTTCGCTCAGCAG	RT-qPCR of *PP_2111* (*aer2*)
qPP_CtpH_F	ATGCAGCAGACCATCGACAT	RT-qPCR of *PP_2120* (*ctpH_PP*)
qPP_CtpH_R	TTTGCCTATCCGTGTGCTGT	RT-qPCR of *PP_2120* (*ctpH_PP*)
qPP_McpA I2_F	AGCAGACCAACCTGCTGGCACTGAAC	RT-qPCR of *PP_2249* (*mcpA*)
qPP_McpA_R5	TGCCATCGATTTCGCCAATG	RT-qPCR of *PP_2249* (*mcpA*)
qPP_2257_F4	ACTGCAATGAACCAGATGGC	RT-qPCR of *PP_2257* (*aer1*)
qPP_2257_R3	CAGGTTGGTTTGCTCGGC	RT-qPCR of *PP_2257* (*aer1*)
qPP_2643_F	GGAGCTGAACAACAAGAGCC	RT-qPCR of *PP_2643* (*pcaY_PP*)
qPP_2643_R	CGTATTCGGCAAAGGTAGCC	RT-qPCR of *PP_2643* (*pcaY_PP*)
qPP_McpP_F	CAAGGCCATAGACACCAGCA	RT-qPCR of *PP_2861* (*mcpP*)
qPP_McpP_R	CGTTCAATGCGGTGAGGTTG	RT-qPCR of *PP_2861* (*mcpP*)
qPP_4521_F	GAAACCATCAAGCAGGGCAA	RT-qPCR of *PP_4521* (*aer3*)
qPP_4521_R	TGATCCGGCCTTGTTCGTAT	RT-qPCR of *PP_4521* (*aer3*)
qPP_McpS_F	ATCCAGTCGATGAACCAGCA	RT-qPCR of *PP_4658* (*mcpS*)
qPP_McpS_R	TTTGCTCTGACACATCACGC	RT-qPCR of *PP_4658* (*mcpS*)
qPP_McpQ_F	CCGTGATGTACTGGGTAGCA	RT-qPCR of *PP_5020* (*mcpQ*)
qPP_McpQ_R	CGTGCTGGTACTGGTTGATG	RT-qPCR of *PP_5020* (*mcpQ*)
qPP_0584_F	ATTTCAAGCAGCTGGGAAGC	RT-qPCR of *PP_0584*
qPP_0584_R	CATGTAGTGCACGCCATTGA	RT-qPCR of *PP_0584*
qPP_0779_F	AGCAAGAAATCCGGCAACTG	RT-qPCR of *PP_0779*
qPP_0779_R	TTGACCATCTCGATCCGTCC	RT-qPCR of *PP_0779*
qPP_1819_F	ACAACCTTGATGTGCTGCAG	RT-qPCR of *PP_1819*
qPP_1819_R	TTGCTCAAGAAACTCGCCAC	RT-qPCR of *PP_1819*
qPP_1940_F	CGTGTTTGAGCCGTTCTTCA	RT-qPCR of *PP_1940*
qPP_1940_R	GAACCGACCCGCTTATTGTC	RT-qPCR of *PP_1940*
qPP_2310_F	CGACAACTAGCCGAGGACAG	RT-qPCR of *PP_2310*
qPP_2310_R	GCTTCGATGGCAGCGTTAAG	RT-qPCR of *PP_2310*
qPP_2823_F	CCAGGCATAACATCGACAGC	RT-qPCR of *PP_2823*
qPP_2823_R	CTTCCAGTTGTTCCATGGCC	RT-qPCR of *PP_2823*
qPP_3414_F	AGCAGATTTCCCAGGAGCTT	RT-qPCR of *PP_3414*
qPP_3414_R	CCCGAATGGTCAGCACAATC	RT-qPCR of *PP_3414*
qPP_3557_F	AATTGGGCAGCAACGAAACC	RT-qPCR of *PP_3557*
qPP_3557_R	GTTGGCCATGTCATGTTCGG	RT-qPCR of *PP_3557*
qPP_3950_F	TACCTGTTCATGGCGCAATC	RT-qPCR of *PP_3950*
qPP_3950_R	ACCGTTCTTGAGGTTTTCGC	RT-qPCR of *PP_3950*
qPP_4888_F	CATTCAGGCGCACATTACCG	RT-qPCR of *PP_4888*
qPP_4888_R	AACAACCCTTCACTCGCCTT	RT-qPCR of *PP_4888*
qPP_4989_F	CATCAACGGCATGGACAACA	RT-qPCR of *PP_4989*
qPP_4989_R	GATGTCGCCGATTTCCTGTG	RT-qPCR of *PP_4989*
qPP_5021_F	TAGGCCTGATCAACGACCTG	RT-qPCR of *PP_5021*
qPP_5021_R	TGGTCGAGGATGTTGCTGAT	RT-qPCR of *PP_5021*
qPP_0387_F	AGGAAATCAACCGTCGCATG	RT-qPCR of *PP_0387* (*rpoD*)
qPP_0387_R	GGTTGGTGTACTTCTTGGCG	RT-qPCR of *PP_0387* (*rpoD*)
qPP_0013_F	CCGCGAAGAGTACAACATCG	RT-qPCR of *PP_0013* (*gyrB*)
qPP_0013_R	ACGGAAGAAGAAGGTCAGCA	RT-qPCR of *PP_0013* (*gyrB*)


### *In vitro* Nucleic Acid Techniques

Plasmid DNA was isolated using the Qiagen spin miniprep kit. For DNA digestion, the manufacturer’s instructions were followed (New England Biolabs and Roche). Separated DNA fragments were recovered from agarose using the Qiagen gel extraction kit. Ligation reactions were performed as described in Sambrook et al. (1989). Competent cells were prepared using calcium chloride and transformations were performed by standard protocols (Sambrook et al., 1989). Phusion^®^ high fidelity DNA polymerase (Thermo Fisher Scientific) was used in the amplification of PCR fragments for cloning.

### Collection of Maize Root Exudates

Corn seeds (*Zea mays* var. Girona) were purchased from Fitó S.A. (Barcelona, Spain). The collection of MRE was carried out as previously indicated (Matilla et al., 2011). Briefly, maize seeds were rinsed and hydrated with sterile deionized water, washed with 70% (v/v) ethanol for 10 min, then with 20% (v/v) bleach for 15 min, followed by thorough rinsing with sterile deionized water. Sterilized seeds were pre-germinated on Phytagel^TM^ agar (Sigma-Aldrich) prepared at 0.3% (w/v) in Plant Nutrient Solution (2.5 mM Ca(NO_3_)_2_, 2.5 mM KNO_3_, 1 mM MgSO_4,_ 0.5 mM KH_2_PO_4_) containing 0.5% (w/v) glucose, 6% (w/v) Fe-citrate, 0.25% (w/v) trace elements (Abril et al., 1989) and incubated at 30°C in the dark for 48 h. Sixteen germinated seeds were transferred into an axenic system with 450 ml of sterile water and allowed to grow at room temperature. After 8 days, the water containing root exudates was collected and vacuum filtrated (0.45 μm cut-off). The resulting solution may also contain non-exuded metabolites derived for example from cell debris or released from root branching points. An aliquot was taken and spread onto solid LB media to check for contamination. Maize root exudates were aliquoted, freeze-dried and stored at -80°C. Before use, the lyophilized exudates were resuspended in sterile water at a 100x concentration (with respect to the 450 ml recipient), centrifuged at 5,000 ×*g* for 10 min and filter-sterilized. MRE at 0.1×, 1×, and 10× concentrations correspond to 0.023, 0.23, and 2.3 g/l.

### Analysis of Root Exudates by Gas Chromatography-Mass Spectrometry (GC-MS)

Ten mg of freeze-dried MRE were resuspended in 120 μl methanol containing 0.2 mg/ml ribitol as internal standard. The solution was dried under nitrogen and derivatized by the addition of 60 μl pyridine containing 20 mg/ml methoxyamine. After brief vortexing, the resulting solution was incubated at 70°C for 120 min. After cooling and a brief centrifugation at 1000 ×*g*, 100 μl of N,O-bis(trimethylsilyl)trifluoroacetamide with 1% (v/v) of the silylation agent trimethylchlorosilane were added and incubated at 70°C for 30 min. Once cooled down, 1 μl samples were injected at 230°C into a 450-GC 240 MS (Varian) Gas chromatography-mass spectrometer using the split mode (100:1). The He carrier gas flow was at 1 ml/min and the capillary chromatography column DB-5MS UI 30 mm × 0.25 mm × 0.25 μm (Agilent Technologies) was used. Column oven ramp was set as follows: 70°C (5 min), to 245°C at 5°C/min, to 310°C at 20°C/min, 310°C (1 min). Electron Impact Ionization was at 70 eV and detection was carried out in Full Scan mode with acquisition between 50 and 600 m/z. Compound identification was achieved using the NIST-08 Standard reference database^1^ and data comparison with compound standards.

### Determination of Chemoreceptor Gene Transcript Levels

Overnight cultures of *P. putida* KT2440 were used to inoculate flasks containing 20 ml M9 medium containing 10 mM glucose to an OD_600_ = 0.05. At an OD_600_ = 0.5, MRE were supplemented from concentrated stock solutions to final concentrations of 0.1×, 1×, and 10×; in parallel a control was supplemented with sterile water. Samples were taken before and 15, 30, and 45 min after root exudate addition. For the determination of gene transcript levels in the presence of one or two chemoeffectors, the same protocol was used, except that ligand solutions to a final concentration of 1 mM were added. RNA extraction and quantitative reverse transcription PCR (RT-qPCR) analyses were performed as described previously (Lopez-Farfan et al., 2017). Briefly, RNA was extracted using the High Pure RNA Isolation Kit (Roche Diagnostics) according to the manufacturer’s instructions and treated with Turbo DNase (Ambion). The extracted RNA was quantified spectrophotometrically using a NanoDrop spectrophotometer (Thermo Scientific). The cDNA was synthesized from 500 ng RNA using the SuperScript^TM^ II Reverse Transcriptase (Invitrogen) and 200 ng of random hexamer primers (Roche). RNA samples not treated with reverse transcriptase were used as negative RT controls. Quantitative PCRs were performed using the iQ^TM^ SYBR^®^ Green supermix (Bio-Rad) and a MyiQ^TM^ thermocycler (Bio-Rad). PCR reactions contained 7.5 μl of 2× SYBR Green supermix, 500 nM of each primer and 1 μl of cDNA in a final volume of 15 μl. All PCR reactions were performed in duplicate. The PCR protocol used was as follows: 95°C for 5 min followed by 35 cycles of 95°C (10 s) and 60°C (30 s) and melting curve analysis from 55 to 95°C, with an increment of 0.5°C/10 s. The primers were designed using the Primer3 Plus software (Untergasser et al., 2012) and are listed in Table 3. Standard curves for each primer pair were generated with serial dilutions of genomic DNA and cDNA to determine PCR efficiency and each product was verified by melting curve analysis. The transcript level data were normalized to that of the reference gene *rpoD* and relative to the control sample using the Bio-Rad iQ5 software. Previous studies have validated *rpoD* as a suitable reference gene in pseudomonads (Savli et al., 2003; Chang et al., 2009). Transcript levels were determined using the relative quantity (ddCt) analysis method (Livak and Schmittgen, 2001). Data are the means and standard deviations of at least three independent experiments. Statistical analyses (*t-*test) were performed using GraphPad software.

### Growth Experiments in the Presence of Exudates

*Pseudomonas putida* KT2440 was grown overnight in M9 medium supplemented with 10 mM glucose. Subsequently, bacterial cultures were diluted into the same medium to an OD_600_ = 0.02 and MRE were added to final concentrations of 0.1×, 1×, and 10×. The same volume of sterile deionized water was added to control cultures. Growth kinetics was determined by following the OD_600_ in a Microbiological Growth Analyzer (Bioscreen, Helsinki, Finland) at 30°C. Data shown are means and standard deviation from three experiments conducted in triplicate.

### Construction of *pp4338* (*cheA2*) Mutant

The mutant defective in *PP_4338* (*cheA2*) was constructed by homologous recombination using a derivative plasmid of the suicide vector pKNG101 (Kaniga et al., 1991). This plasmid, named pMAMV290 (Table 2), carried a mutant allele for the replacement of the gene in the chromosome and was transferred to *P. putida* KT2440R by triparental conjugation using *E. coli* CC118λ*pir* and *E. coli* HB101 (pRK600) as helper. For the construction of the final mutant, sucrose was added to a final concentration of 10% (w/v) to select derivatives that had undergone a second cross-over event during marker exchange mutagenesis. The resulting mutants were confirmed by PCR and DNA sequencing.

### Quantitative Capillary Chemotaxis Assays Toward MRE

Overnight cultures of *P. putida* KT2440R strains were diluted to an OD_660_ of 0.075 in M9 minimal medium supplemented with 6 mg l^-1^ Fe-citrate, trace elements (Abril et al., 1989) and 10 mM sodium benzoate as carbon source, and grown at 30°C with orbital shaking (200 rpm). At an OD_660_ of ∼0.4, cultures were centrifuged at 1,700 ×*g* for 5 min and the resulting pellet was washed twice with chemotaxis buffer [50 mM potassium phosphate, 20 mM EDTA, 0.05% (v/v) glycerol, pH 7.0]. Subsequently, the cells were resuspended in the same buffer, adjusted to an OD_660_ = 0.1 and 230 μl aliquots of the bacterial cultures were placed into 96-well plates. Subsequently, one-microliter capillary tubes (Microcaps, Drummond Scientific, Ref. P1424) were heat-sealed at one end and filled with either the chemotaxis buffer (negative control), different concentrations of MRE or 0.2% (w/v) casamino acids. The capillaries were immersed into the bacterial suspensions at its open end and after 30 min at room temperature; capillaries were removed from the bacterial suspensions, rinsed with sterile water and the content expelled into 1 ml of M9 minimal medium. Serial dilutions were plated onto M9 minimal medium supplemented with 15 mM glucose as carbon source. The number of colony forming units was determined after overnight incubation. In all cases, data were corrected with the number of cells that swam into buffer containing capillaries.

### Competitive Root Colonization Assays

Maize seeds were sterilized, germinated and inoculated as described previously (Matilla et al., 2007). Briefly, sterile seeds were incubated at 30°C with a 5 × 10^6^ CFU/ml 1:1 mixture of KT2440RTn*7*-Sm (wild type) and a mutant strain defective in the corresponding *cheA* gene for 30 min. Thereafter, seeds were rinsed with sterile deionized water and planted in 50 ml Sterilin tubes containing 40 g of sterile washed silica sand. Plants were maintained at 24°C with a daily light period of 16 h. After 6 days, bacterial cells were recovered from the rhizosphere. Briefly, plant roots were collected, shaken vigorously to discard loosely attached silica sand and introduced into 50 ml sterile tubes containing 10 ml of M9 salts medium (Sambrook et al., 1989) and 4 g of glass beads (3 mm of diameter). Tubes were vortexed for 1 min and serial dilutions were plated onto LB-agar medium supplemented with streptomycin or kanamycin to select the wt or the mutant strains, respectively.

### Construction of Expression Plasmids for PP4888-LBD

The DNA fragments encoding the LBDs of PP4888 of *P. putida* KT2440 were amplified by PCR using genomic DNA and primers listed in Table 3. The resulting product was digested and cloned into the expression plasmid pET28b(+) to create pPP4888. These plasmids were verified by DNA sequencing.

### Overexpression and Purification of Proteins

The different recombinant ligand binding domains were expressed in *E. coli* and purified by metal affinity chromatography using standard conditions.

### Thermal Shift Assays

Assays were performed as reported in Fernandez et al. (2018) using a final DIBOA concentration of 1 mM.

### Isothermal Titration Calorimetry

Experiments were conducted on a VP-microcalorimeter (Microcal, Amherst, MA, United States) at 15°C. PP4888-LBD was dialyzed into 20 mM Hepes, 150 mM NaCl, 10% (v/v) glycerol, pH 7.4, adjusted to 50 μM and titrated with 0.5 mM of DIBOA prepared in dialysis buffer.

### Colony-Based c-di-GMP Reporter Assays

Fluorescence intensity analyses using the c-di-GMP biosensor pCdrA::*gfp*^C^ were carried out to determine the intracellular levels of the second messenger c-di-GMP. To this end, the reporter plasmid pCdrA::*gfp*^C^ was transformed into KT2440R strains by electroporation. Subsequently, overnight bacterial cultures of the strains to be tested were adjusted to an OD_660_ = 0.05 and 20 μl aliquots were placed onto LB-agar plates containing the appropriate antibiotics. Following incubation at 30°C for 48 h, fluorescence intensities were recorded using a Leica M165 FC stereomicroscope. Fluorescence was visualized employing a GFP filter set (emission/excitation filter 470/525 nm). Pictures were taken using Leica Application Suite software using different exposure times.

### Biofilm Formation Assays

Biofilm assays in borosilicate glass tubes were carried out with bacterial cultures grown in LB medium. Overnight cultures were adjusted to an OD_660_ = 0.05 and borosilicate tubes containing 1 ml of these cultures were incubated at 30°C under orbital shaking (40 rpm) in a Stuart SB3 tube rotator (Cole-Parmer). At the indicated times, bacterial cultures were removed, tubes rinsed three times with water and biofilms stained with 3 ml of crystal violet (0.5%, w/v) for 15 min at room temperature. Staining solution was discarded and tubes were rinsed three times with water. Biofilm formation was quantified by solubilizing the dye with 70% (v/v) ethanol and measuring the absorbance at 540 nm.

### Swimming Motility Assays

Overnight cultures were adjusted to an OD_660_ = 1 and 2 μl of these cultures were spotted onto LB-Difco agar (0.3% [w/v]) plates and incubated at 30°C.

## Results

### The Effect of Maize Root Exudates on Chemoreceptor Gene Transcript Levels

Root exudates were obtained from maize and analyzed by gas chromatography-mass spectrometry (GC-MS) to get information mainly on their composition in primary metabolites (Supplementary Figure S2). Compounds detected are listed in Supplementary Table S1 and include different sugars, sugar acids, amino acids and organic acids (Supplementary Table S1). To assess the effect of MRE on chemoreceptor gene transcript levels, KT2440 was grown in M9 minimal medium supplemented with 10 mM glucose. At an OD_660_ of 0.5, MRE were added to the culture at three different concentrations: 1× corresponds to the MRE concentration in the 450 ml recipient that contained 16 germinated maize seedlings for 8 days (see section “Materials and Methods” for further details), whereas the 0.1× and 10× concentrations are 10-fold diluted or concentrated samples thereof. Chemoreceptor gene transcript levels were quantified prior and 15, 30, or 45 min after MRE addition. As a control, the transcript levels of the housekeeping gene *gyrB* were measured in parallel.

**FIGURE 1:**
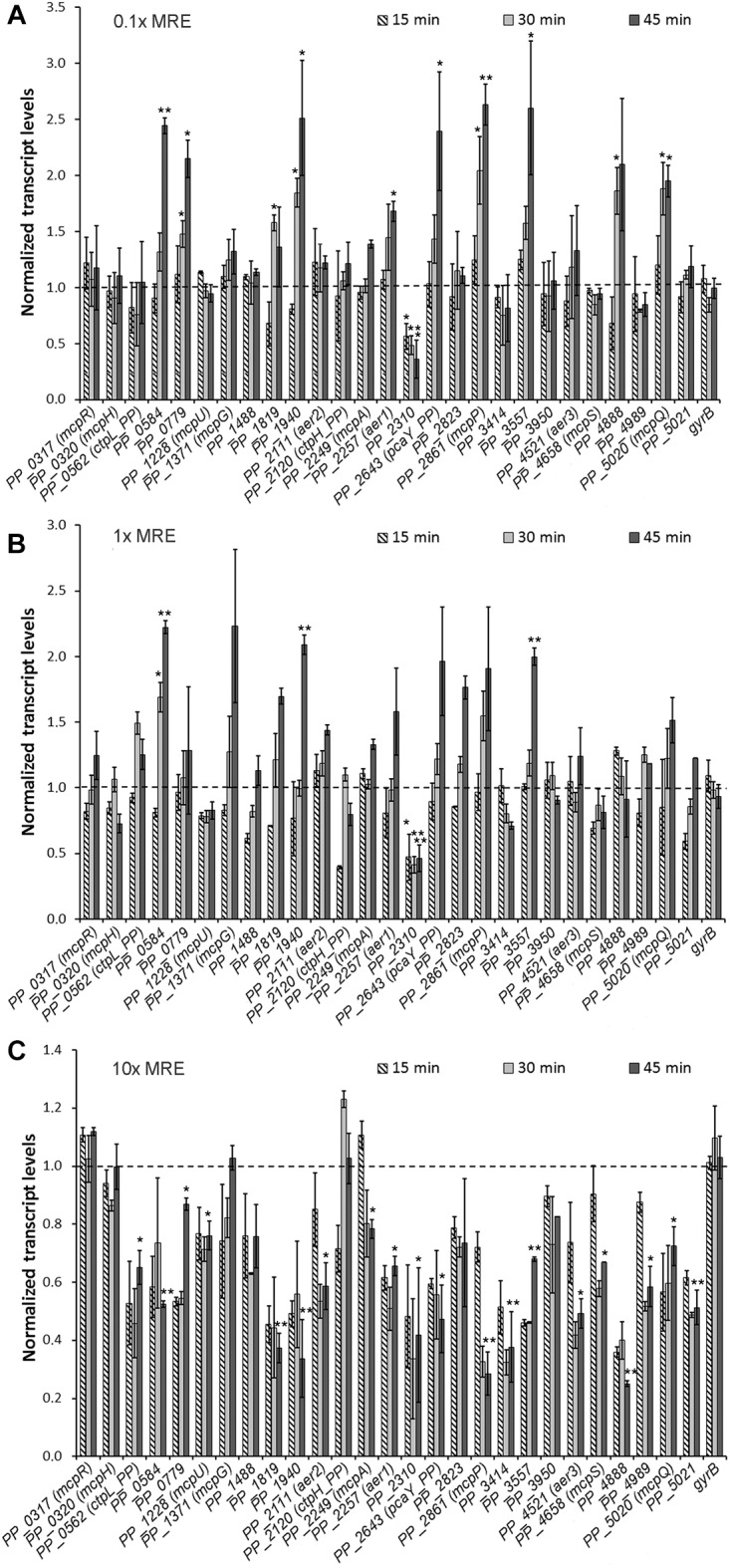
Transcript levels of the 27 chemoreceptor genes in response to different maize root exudate (MRE) concentrations. *Pseudomonas putida* KT2440 cells were grown to an OD_600_ = 0.5 in M9 medium containing 10 mM glucose as carbon source and MRE were added at a concentration of 0.1× **(A)**, 1× **(B)**, and 10× **(C)**. Samples were taken 15, 30, and 45 min after MRE addition. Results were normalized with the reference gene *rpoD* and relative to the control sample (M9 + glucose medium). Data are the means and standard deviations from at least three independent experiments. The maximal standard deviations for the control measurements were of 0.16. ^∗^*P* < 0.05, ^∗∗^*P* < 0.01 (by Student’s *t*-test). For clarity reasons statistical relevance in **(C)** is only indicated for the 45 min measurement.

As shown in Figure 1A, an at least twofold induction of 8 chemoreceptor gene transcripts was noted at the 0.1× MRE concentration. The function of three of these receptors has been established since McpP, McpQ and PcaY_PP were found to bind and mediate chemoattraction to C2/3-carboxylic acids (Garcia et al., 2015), citrate (Martin-Mora et al., 2016) and C6 ring cyclic carboxylic acids (Fernandez et al., 2017), respectively. In the case of McpP and PcaY_PP, previous studies have shown that the cognate chemoeffectors increase chemoreceptor gene transcript levels, whereas citrate had no effect on *mcpQ* transcript levels (Lopez-Farfan et al., 2017). Another chemoreceptor gene induced by 0.1× MRE was *PP_4888* (Figure 1A), which agrees with the study by Neal et al. (2012). The authors of this report show that MRE contain a significant amount of benzoxazinoids that attract *P. putida* chemotactically to the rhizosphere. To verify whether PP_4888 binds benzoxazinoids, we have cloned the DNA sequence encoding the LBD of this receptor into an expression plasmid and purified the recombinant protein from *E. coli* cultures. However, microcalorimetric titrations and thermal shift assays with DIBOA (2,4-dihydroxy-1,4-benzoxazin-3-one) did not reveal binding indicating that PP_4888 does not bind this ligand directly. The remaining four chemoreceptor genes with at least twofold increased transcript levels, *PP_0584*, *PP_0779*, *PP_1940*, and *PP_3557*, are of unknown function. Additionally, two chemoreceptor genes, *PP_1819* and *PP_2257*, showed a statistically significant increase in their transcript levels (Figure 1A). The function of PP_1819 remains unknown but PP_2257 is predicted to be an energy taxis receptor. Of note, exposition to 0.1× MRE reduced transcript levels of only one chemoreceptor gene, *PP_2310* (Figure 1A).

At a 1× MRE concentration a similar set of chemoreceptor genes showed increased transcript levels. However, the magnitude of induction was below that seen at the 0.1× concentration and a statistically significant increase was only observed for three receptors genes (Figure 1B). Interestingly, at a 10× MRE concentration, twenty chemoreceptor transcript levels showed statistically relevant reductions (Figure 1C). These include the ten receptors genes that were upregulated at the 0.1× MRE concentration.

On the other hand, genes encoding functionally annotated chemoreceptors including McpR for organic acids, McpH for purines, McpG for GABA and CtpH_PP for inorganic phosphate were little affected by 10× MRE (Figure 1C). Previous studies have shown that the cognate ligands do not alter *mcpR* and *mcpG* transcript levels whereas purines and inorganic phosphate either up- or downregulated, respectively, the transcript levels of their receptor genes (Lopez-Farfan et al., 2017). At all three MRE concentrations gene transcript levels of the *gyrB* control gene were unchanged.

### Study of Chemoreceptor Gene Transcript Levels in the Presence of Ligand Mixtures

The magnitude of MRE mediated changes in chemoreceptor gene transcript levels were well below that observed in our previous study where we have evaluated the effect of 1 mM chemoeffectors on the transcript levels of their cognate chemoreceptor genes (Lopez-Farfan et al., 2017). For example, the cognate chemoeffectors acetate and 4-hydroxybenzoate enhanced *mcpP* and *pcaY_PP* transcript levels by factors of 22 and 35, respectively (Lopez-Farfan et al., 2017). This contrasts with the present data where MRE enhanced transcript levels of both chemoreceptors genes approximately 2.5-fold.

**FIGURE 2:**
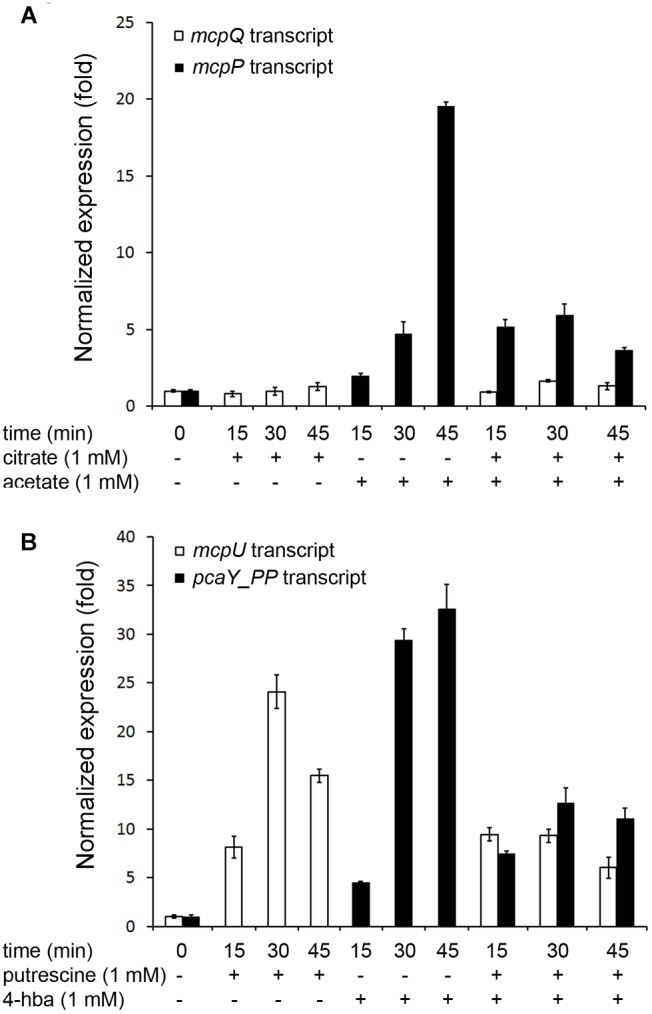
Chemoreceptor gene transcript levels in absence and the presence of single or two simultaneously added chemoattractants. KT2440 cultures were grown to an OD_600_ = 0.5 (time 0) and a single or two chemoattractants were added to final concentrations of 1 mM for each compound. Samples were taken at the times indicated and the transcript levels of the chemoreceptor genes *mcpQ* and *mcpP* **(A)** or *mcpU* and *pcaY_PP* **(B)** were determined by RT-qPCR. 4-hba: 4-hydroxybenzoate. Data are means and standard deviations from three experiments.

To cast light into this issue, we quantified the chemoreceptor gene transcript levels in cultures that contain either a single or two chemoeffectors, each at final concentrations of 1 mM. As shown in Figure 2A, the addition of 1 mM citrate did not increase *mcpQ* gene transcript levels, whereas in another culture the addition of 1 mM acetate enhanced *mcpP* transcript levels 20-fold, confirming previous studies (Lopez-Farfan et al., 2017). However, in a culture to which both, citrate and acetate have been added, the increase in *mcpP* transcript levels was only around sixfold, whereas that of *mcpQ* was unchanged. Additionally, in a similar experiment, changes in the transcript levels of *mcpU* and *pcaY_PP* by their cognate chemoeffectors was studied (Figure 2B). Putrescine and 4-hydroxybenzoate on their own (at 1 mM) increased *mcpU* and *pcaY_PP* transcript levels 24- and 32-fold, respectively. However, co-addition of both ligands (both at 1 mM) increased transcript levels of their receptors genes only 6–12-fold.

Given the disparity in the changes of chemoreceptor genes transcript levels in the presence or absence of single or multiple chemoeffectors, our results indicate that there appears to be an intrinsic capacity of the cell to modulate receptor gene transcript levels; capacity that is shared among the individual receptors genes in the presence of multiple ligands.

### Different Root Exudate Concentrations Do Not Alter Significantly Growth Kinetics

We were intrigued by the finding that at the 10× MRE concentration almost all chemoreceptor genes showed reduced transcript levels. MRE were found to contain significant amounts of benzoxazinoids like DIMBOA or DIBOA (Neal et al., 2012) that are plant secondary metabolites with a potent antibiotic activity (Wouters et al., 2016). To assess the effect of MRE on growth kinetics, we have recorded growth curves in M9 medium with 10 mM glucose as carbon source (control) and in this medium supplemented with different MRE concentrations. As shown in Supplementary Figure S3, the addition of 0.1× MRE did not alter growth kinetics significantly and only a slight reduction in the lag time was observed in the presence of 1 and 10× MRE. These differences in growth kinetics are thus unlikely to account for the differences in transcript levels seen at the 10× MRE concentration.

### Chemotactic Responses Occur Only at Elevated Root Exudate Concentration

**FIGURE 3:**
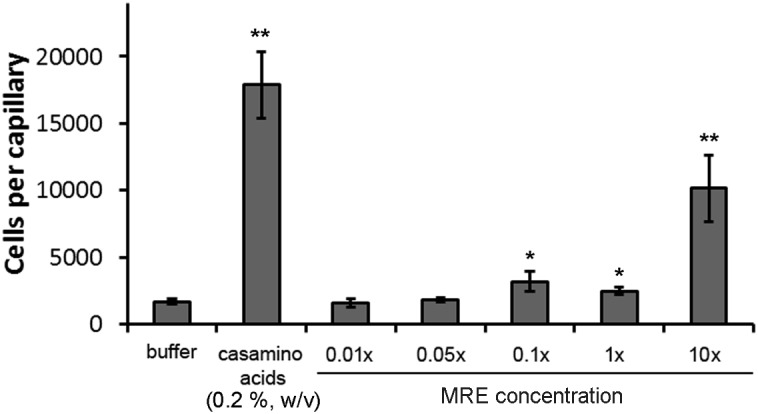
Quantitative capillary chemotaxis assays of *P. putida* KT2440R to different maize root exudate concentrations. Chemotaxis to buffer and a 0.2% (w/v) casamino acid solution are shown as controls. Data are means and standard deviations from three individual experiments conducted in triplicates. ^∗^*P* < 0.05, ^∗∗^*P* < 0.01 (by Student’s *t*-test).

Interestingly, above data show that the most significant increase in chemoreceptor gene transcript levels occurred at the lowest MRE concentration. Since most of these receptors mediate chemotaxis, we assessed the chemotactic behavior of *P. putida* KT2440 to different MRE concentrations. To this end, we have carried out quantitative capillary chemotaxis assays, which is the traditional reference assay to quantify chemotaxis. In this assay, the chemoattractant is placed into a capillary that is then submerged into the bacterial suspension. After a contact time of 30 min, the capillaries are withdrawn, emptied and the number of colony forming units is determined. The expected strong chemotaxis was observed for a 0.2% (w/v) casamino acid solution that served as a positive control. No responses were noted for 0.01 and 0.05× MRE, whereas only minor chemotaxis was observed for the 0.1 and 1× concentrations (Figure 3). In contrast, MRE concentrations of 10× induced significant taxis (Figure 3).

### Effect of Root Exudates on c-di-GMP Levels, Biofilm Formation and Swimming Motility

**FIGURE 4:**
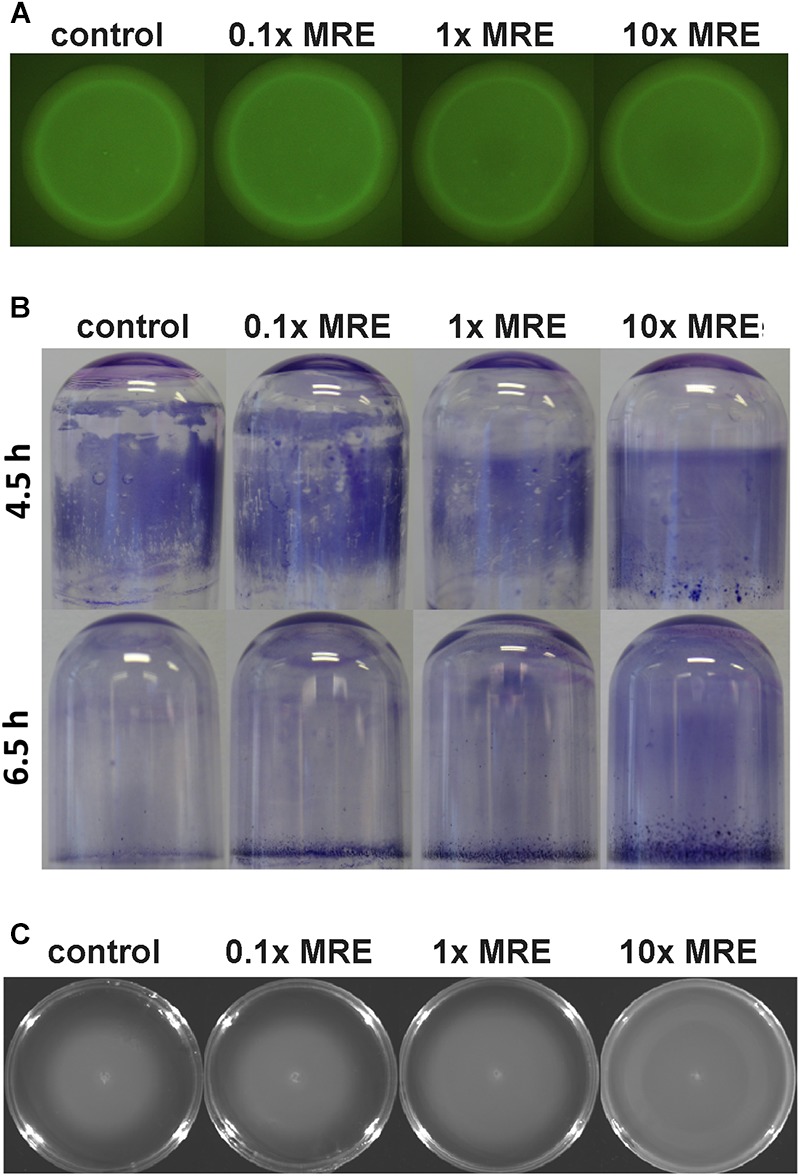
The effect of different root exudate concentrations on c-di-GMP levels, biofilm formation and motility of *P. putida* KT2440R. Cells were grown as liquid cultures **(B)** or on agar plates **(A,C)** in minimal medium supplemented with 10 mM glucose in the absence and presence of different MRE concentrations, which are the same conditions as used for RT-qPCR studies. **(A)** Fluorescence intensity of colonies harboring the c-di-GMP biosensor plasmid pCdrA::*gfp*^C^. **(B)** Biofilm formation on borosilicate tubes during growth with orbital shaking at 40 r.p.m. **(C)** Swimming motility after an 16 h incubation at 30°C. Each of these assays was conducted three times and representative images are shown.

We conducted a number of studies to characterize the effect of MRE on different aspects of bacterial physiology. In all experiments cells were cultured in conditions that have been used for the quantification of chemoreceptor transcript levels, namely in minimal medium with glucose as carbon source in the absence and presence of different MRE concentrations. As mentioned above, a reduction of most chemoreceptor gene transcripts was observed at a 10x MRE concentration, suggesting that this may be due to a general regulatory mechanism. To assess whether this reduction may be caused by an alteration in the c-di-GMP levels, the plasmid pCdrA::*gfp*^C^ harboring a c-di-GMP biosensor was introduced into *P. putida* KT2440 that was grown on plates containing different MRE concentrations. Since the output of this biosensor is the synthesis of the green fluorescent protein, individual colonies were analyzed by fluorescence microscopy. However, as shown in Figure 4A, the exposure of the bacterium to different MRE concentrations did not result in significant changes in the c-di-GMP level.

Subsequent studies were aimed at elucidating the effect of different MRE concentrations on biofilm formation and dispersal. Biofilms were grown in borosilicate glass tubes under constant shaking. We noted that the biofilm formed in the presence of MRE was more homogeneous and more tightly attached to the glass surface (Figure 4B). This tightness of association was also reflected in a significant delay of biofilm dispersion with increasing MRE concentrations (Figure 4B). In subsequent experiments the swimming motility was assessed on 0.3% (w/v) agar plates containing different MRE concentrations. As shown in Figure 4C an increase in bacterial motility was observed with increasing MRE concentration.

### Involvement of Different Chemosensory Pathways in Chemotaxis to Root Exudates

**FIGURE 5:**
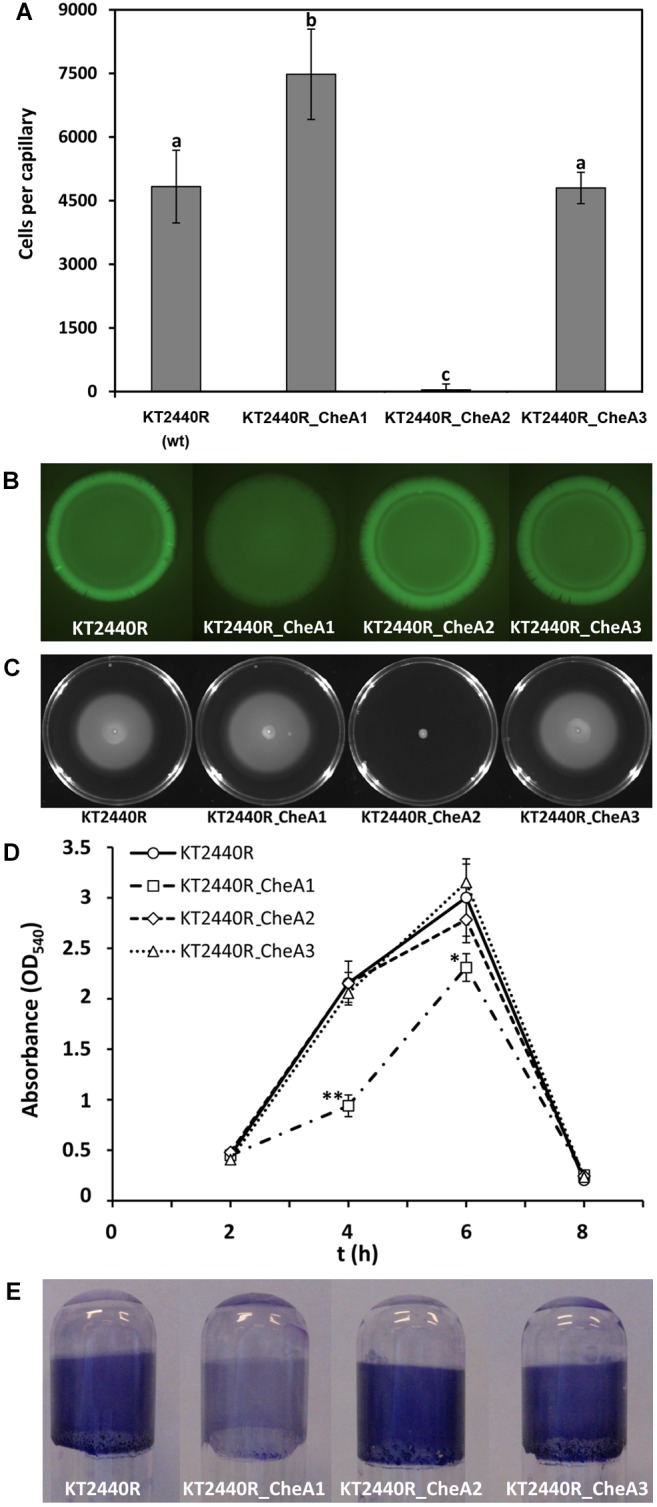
Functional analyses of the three CheA paralogs in *P. putida* KT2440. **(A)** Quantitative capillary chemotaxis assays to 10× MRE. Data were corrected with the number of cells that swam into buffer containing capillaries (from left to right, 1960 ± 339; 2093 ± 191; 2133 ± 280; 1980 ± 254). Data are the means and standard deviations from three biological replicates conducted in triplicate. Bars with the same letter are not significantly different (*P*-value < 0.05; by Student’s *t*-test). **(B)** Fluorescence intensity of *P. putida* KT2440R strains harboring the c-di-GMP biosensor plasmid pCdrA::*gfp*^C^. Experiments were conducted on LB agar plates. The assays were repeated three times and representative results are shown. **(C)** Swim plate motility assays. Shown is a representative experiment. Means and standard deviations of halo diameters from three biological replicates are shown in Supplementary Figure S6. **(D)** Quantification of biofilms stained with crystal violet. Data are means and standard deviations of three biological replicates. ^∗^*P* < 0.05, ^∗∗^*P* < 0.01, Student’s *t*-test of KT2440R_CheA1 with respect to the wt strain. **(E)** Biofilm formation in borosilicate glass tubes after 4 h of growth in LB medium with orbital shaking.

KT2440 has three CheA paralogs (Supplementary Figure S1) that are likely to form part of three pathways. To investigate the role of these three potential pathways in the chemotaxis to MRE, quantitative capillary chemotaxis assays to 10× MRE were conducted using mutants of *cheA1*, *cheA2*, and *cheA3*. As shown in Figure 5A, mutation in *cheA1* increased the magnitude of chemotaxis, whereas mutation of *cheA2* abolished chemotaxis, which is consistent with the notion the CheA2 forms part of the *che* chemotaxis pathway. Chemotaxis of the *cheA3* mutant was indistinguishable from that of the wt strain.

To assess whether this increase in chemotaxis in the *cheA1* mutant is due to changes in the c-di-GMP levels, plasmid pCdrA::*gfp*^C^ was introduced into the wt and the three *cheA* mutants and the resulting strains were analyzed by fluorescence microscopy. As shown in Figure 5B, c-di-GMP levels were significantly reduced in the *cheA1* mutant, whereas those of the other two *cheA* mutants were comparable to the wt. In a number of bacteria including KT2440 it has been shown that low c-di-GMP levels correlate with an increase in motility (Matilla et al., 2011; Romling et al., 2013). Therefore, the reduction in the c-di-GMP levels is likely to account for the slight, but reproducible increase in swimming motility observed for the *cheA1* mutant (Figure 5C). We have subsequently investigated the contribution of the three CheA paralogs to biofilm formation. The kinetics of biofilm formation of the *cheA1* mutant was distinct from that of the other strains, since this mutant formed significantly less biofilm after 4 and 6 h (Figure 5D). This difference was particularly pronounced after 4 h (Figure 5E). These data suggest the existence of functional *wsp_PP* and *che_PP* pathways, of which the latter is involved in root exudate chemotaxis.

### Involvement of Different Pathways in the Competitive Colonization of Maize Roots

**FIGURE 6:**
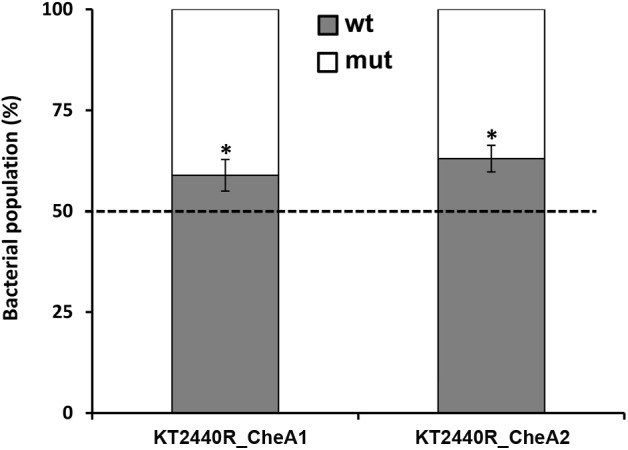
Competitive root colonization of *Pseudomonas putida* KT2440RTn*7*-Sm and mutants in different *cheA* genes. Shown is the relative abundance of mutant and wild type strains recovered from the rhizosphere of maize plants. Proportion of wild type and mutant strains in the initial inocula was 50 ± 3% (Supplementary Figure S5). Data are the means and standard deviations of six plants. ^∗^*P* < 0.01, Student’s *t*-test of mutant strains with respect to the wt strain.

It was shown previously that a mutant in *cheA3* (*PP_4988*) exhibited reduced competitive colonization fitness to colonize maize roots as compared to the wt strain (Matilla et al., 2007). To determine the role of the remaining two pathways in the root colonization ability of KT2440, we have conducted competitive root colonization assays with mutants in *cheA1* and *cheA2* with the wt strain. Initial control experiments showed that the different strains showed similar growth kinetics (Supplementary Figure S4). In these assays equal amounts of wt and mutant strains with different antibiotics susceptibilities are used to inoculate maize roots. Supplementary Figure S5 shows another control experiment illustrating that the bacterial mixtures used for inoculation indeed contain the same amount of wt and mutant strains. Six days following inoculation bacteria are recovered and identified by plating out on media containing different antibiotics. As shown in Figure 6, both mutants were slightly less competitive in colonizing maize roots indicating that the *che_PP* as well as *wsp_PP* pathways play significant roles in root colonization.

## Discussion

Using *P. putida* KT2440 as model bacterium, we present a study of the effect of root exudates on the transcript levels of the whole repertoire of chemoreceptor genes of a bacterium. Whereas at a distance to the root, exudates enhance chemoreceptor transcript levels promoting in turn chemotaxis, this process is reversed in root vicinity, where the necessity of root chemotaxis may be minor. A major conclusion of this work is that there is a temporal dimension to the effect of root exudates in the rhizosphere that has to be taken into account in studies to investigate plant–bacteria interaction. There are a number of studies that have investigated the impact of root exudates on the transcript levels of chemotaxis and motility genes (de Weert et al., 2002; Oku et al., 2012; Webb et al., 2016). However, data reported are contrasting, which is best illustrated by research on the influence of MRE on the gene transcript levels of two very similar plant growth promoting rhizobacteria, namely *Bacillus atrophaeus* UCMB-5137 and *B. amyloliquefaciens* FZB42. Whereas chemotaxis and motility genes were downregulated by MRE in the former strain (Mwita et al., 2016), they were upregulated in the latter strain (Fan et al., 2012).

What are thus the mechanisms responsible for these alterations in transcript levels? A significant number of chemoeffectors were shown to increase transcript levels of their cognate chemoreceptors, whereas a reduction was solely observed for the inorganic phosphate responsive receptors (Lopez-Farfan et al., 2017). It can thus be hypothesized that part of the increases observed at low MRE are due to the action of cognate ligands. However, the fact that almost all chemoreceptor transcripts were reduced at high MRE concentration suggests the existence of a more general mechanism, which may potentially be linked to the MRE induced changes in biofilm formation (Figure 4B). In accordance with this, previous results in our group established a link between the absence of chemotaxis and biofilm formation since the mutation of specific chemoreceptor genes resulted in an increased biofilm formation by KT2440 (Corral-Lugo et al., 2016). The second messenger c-di-GMP is the key determinant to define the balance between planktonic and sessile lifestyles (Romling et al., 2013) and in KT2440 it was found to enhance biofilm formation (Matilla et al., 2011; Ramos-Gonzalez et al., 2016). Remarkably, several studies have revealed that this second messenger can interfere with chemotactic processes, for example, by modulating the methylation state of chemoreceptors (Xu et al., 2016; Nesper et al., 2017) or by increasing swimming velocity through direct binding to chemoreceptor proteins (Russell et al., 2013). Therefore, we hypothesized that the general mechanism that modulates chemoreceptor transcripts levels may be based on alterations of the c-di-GMP level. However, our experiments with a c-di-GMP biosensor did not provide any evidence for MRE mediated changes in the level of this second messenger (Figure 4A) and further research is thus necessary to decipher the molecular mechanisms.

KT2440 has three paralogs of most chemosensory signaling proteins (Supplementary Figure S1), which is consistent with the notion that the 27 chemoreceptors feed into three different pathways, in a way comparable to that in *P. aeruginosa* (Ortega et al., 2017). Inactivation of the *cheA2* gene abolished chemotaxis to MRE (Figure 5A), which agrees with the finding that the *cheR2* mutant was also largely impaired in chemotaxis (Garcia-Fontana et al., 2013). Both proteins thus form part of a chemotaxis pathway similar to the *P. aeruginosa* *che* chemotaxis pathway (Ortega et al., 2017). In contrast, inactivation of *cheA1* reduces c-di-GMP levels and increased chemotaxis to MRE (Figure 5A,B), which is likely due to the slight but reproducible increase in motility of this mutant (Figure 5C and Supplementary Figure S6). This mutant was also largely impaired in biofilm formation (Figure 5D,E), and a similar phenotype has been observed previously for the *cheR2* mutant (Garcia-Fontana et al., 2013). These observations and the similarity of the corresponding gene cluster to *P. aeruginosa* indicates that both proteins form part of a signaling cascade homologous to the *wsp* pathway (Hickman et al., 2005). The output of this pathway is not directly related to chemotaxis but consists in a modulation of c-di-GMP levels potentially incorporating signals that stimulate the chemoreceptor PP1488 (homologous to *P. aeruginosa* WspA) (Hickman et al., 2005; O’Connor et al., 2012). Taken together, data indicate the existence of functional *che* and *wsp* pathways in KT2440. Importantly, signaling through both pathways is important for efficient root colonization since for both *cheA* mutants a discrete but reproducible reduction in competitive root colonization was observed (Figure 6). This suggests that chemotaxis to root exudates as well as an adjustment of c-di-GMP levels are two different, but interconnected processes that are required for efficient root colonization. Taken together, this work provides insight into regulatory mechanisms that occur in the rhizosphere. Future work will show whether the observed, dose-dependent effect of root exudates is also common to other rhizobacteria.

## Author Contributions

DL-F, JR-D, and MM conducted the research and analyzed the data. MM and TK planned the research, analyzed the data, and wrote the manuscript.

## Conflict of Interest Statement

The authors declare that the research was conducted in the absence of any commercial or financial relationships that could be construed as a potential conflict of interest.

## Footnotes

1 https://www.nist.gov/srd/updated-database
