# Supplementary material for: Concentration Dependent Effect of Plant Root Exudates on the Chemosensory Systems of Pseudomonas putida KT2440
Source: Front Microbiol. 2019 Jan 30;10:78. doi: 10.3389/fmicb.2019.00078 (PMC6363813; doi:10.3389/fmicb.2019.00078)
Supplement: Supplementary file 1 [file Data_Sheet_1.docx]

Supplementary Material

to

**Concentration dependent effect of plant root exudates on the chemosensory systems of *Pseudomonas putida* KT2440**

by

Diana López-Farfán, José A. Reyes-Darias, Miguel A. Matilla and Tino Krell


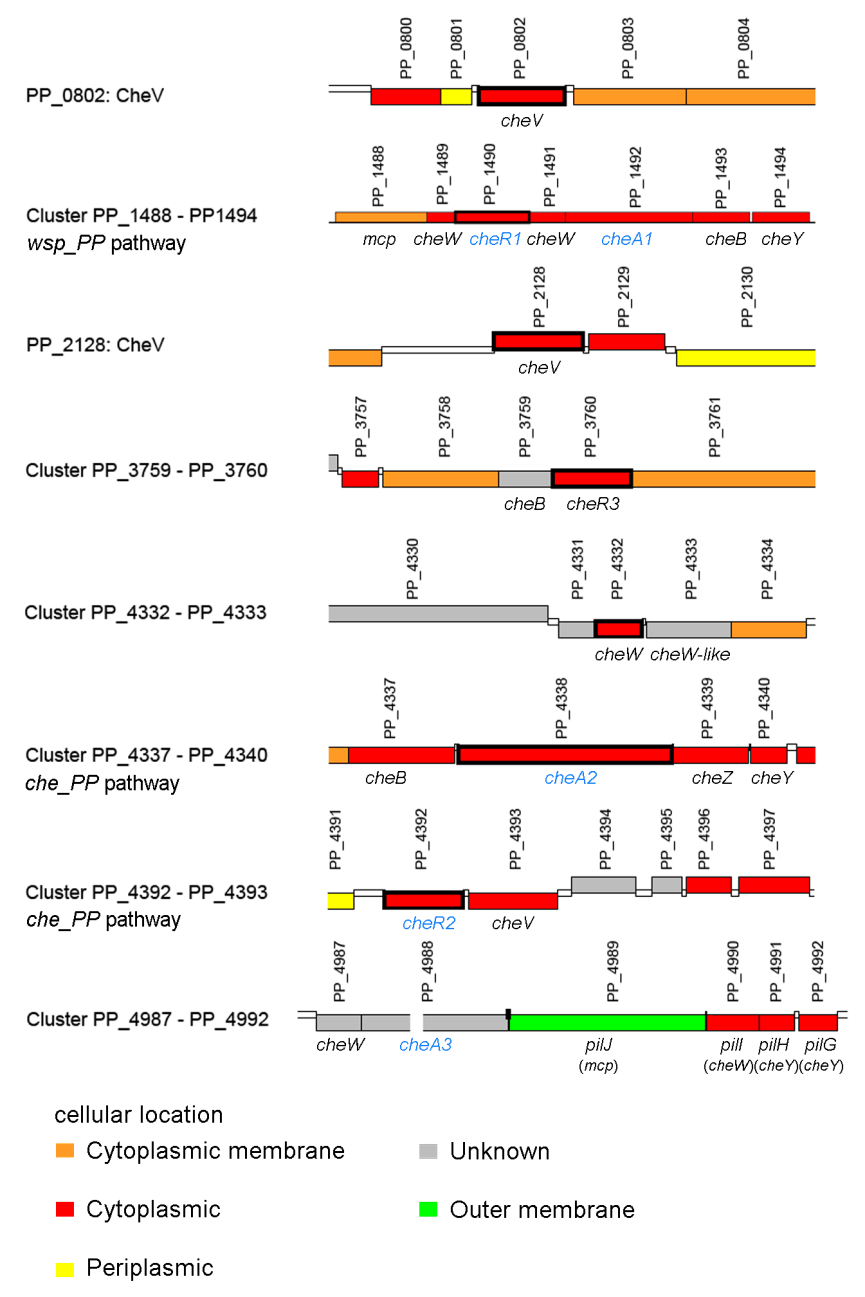


**Supp. Fig. 1) Genes encoding signaling proteins of chemosensory pathways in *P. putida* KT2440**. Modified version of figure published in García-Fontana *et al*. (2013) J Biol Chem 288:18987-99. This Figure was generated based on information in the Pseudomonas Genome Database (<http://www.pseudomonas.com/>). The corresponding chemosensory pathways of some of the gene clusters are indicated. The colors indicate the predicted cellular location of the respective proteins according to Pseudomonas.com.


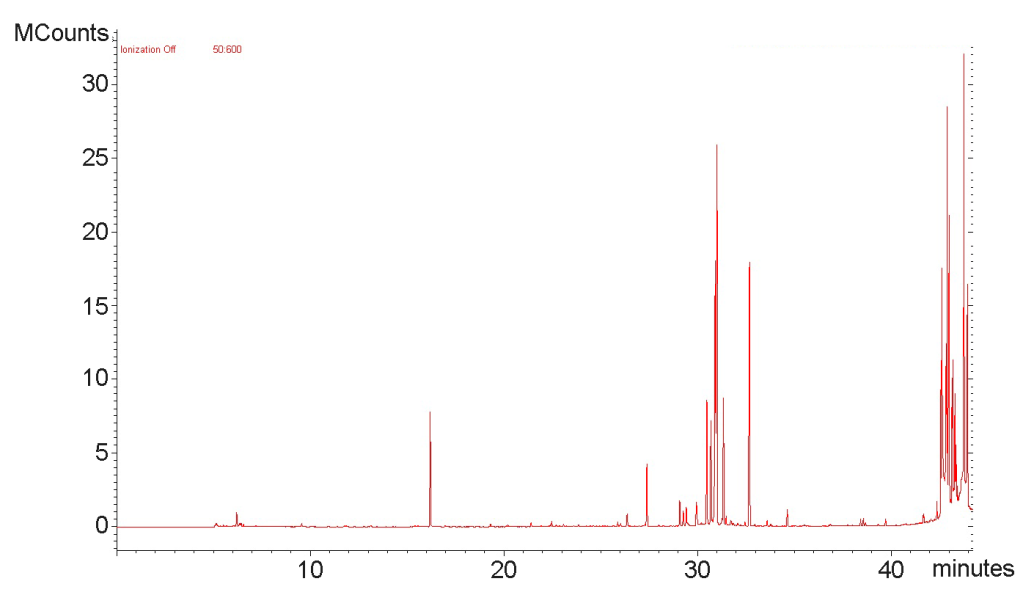


**Supp. Fig. 2) Gas chromatography-mass spectrometry chromatogram of MRE.** Compounds identified are listed in Supp. Table 1.


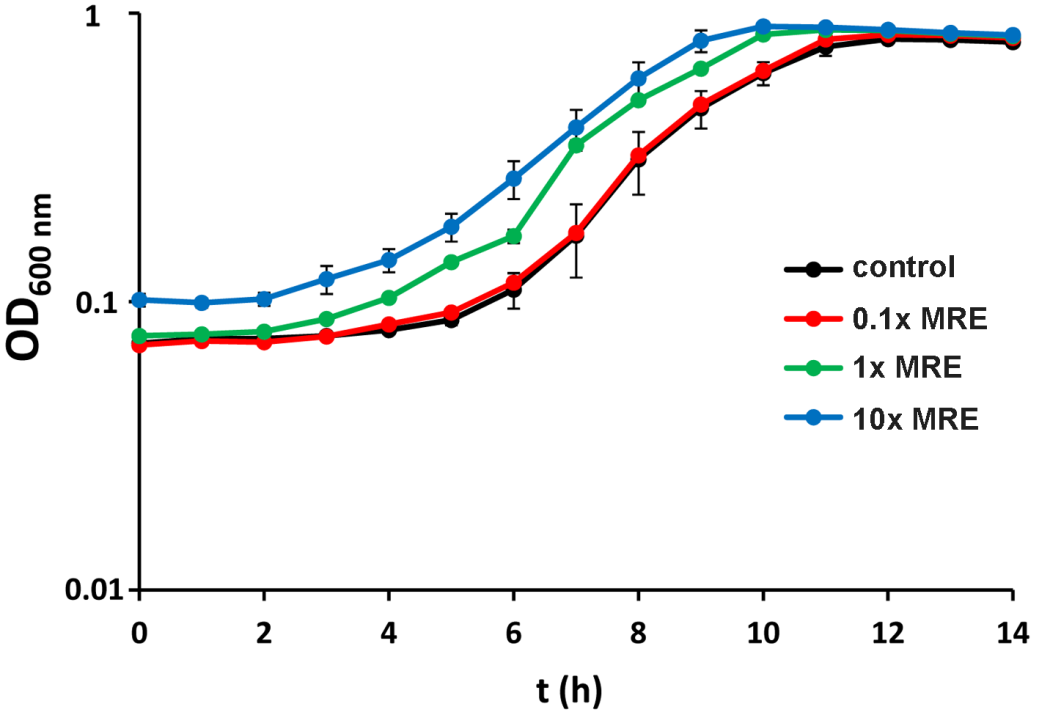


**Supp. Fig. 3) The effect of different maize root exudate concentrations on bacterial growth.** Growth kinetics of *P. putida* KT2440 in M9 medium containing 10 mM glucose in the absence or presence of different MRE concentrations. Doubling times in exponential phase of growth were 104 ± 10 min, 97 ± 1 min, 104 ± 3 min and 105 ± 2 min for control, 0.1x MRE, 1x MRE and 10x MRE, respectively. Data are the means and standard deviations of three biological replicates.

**
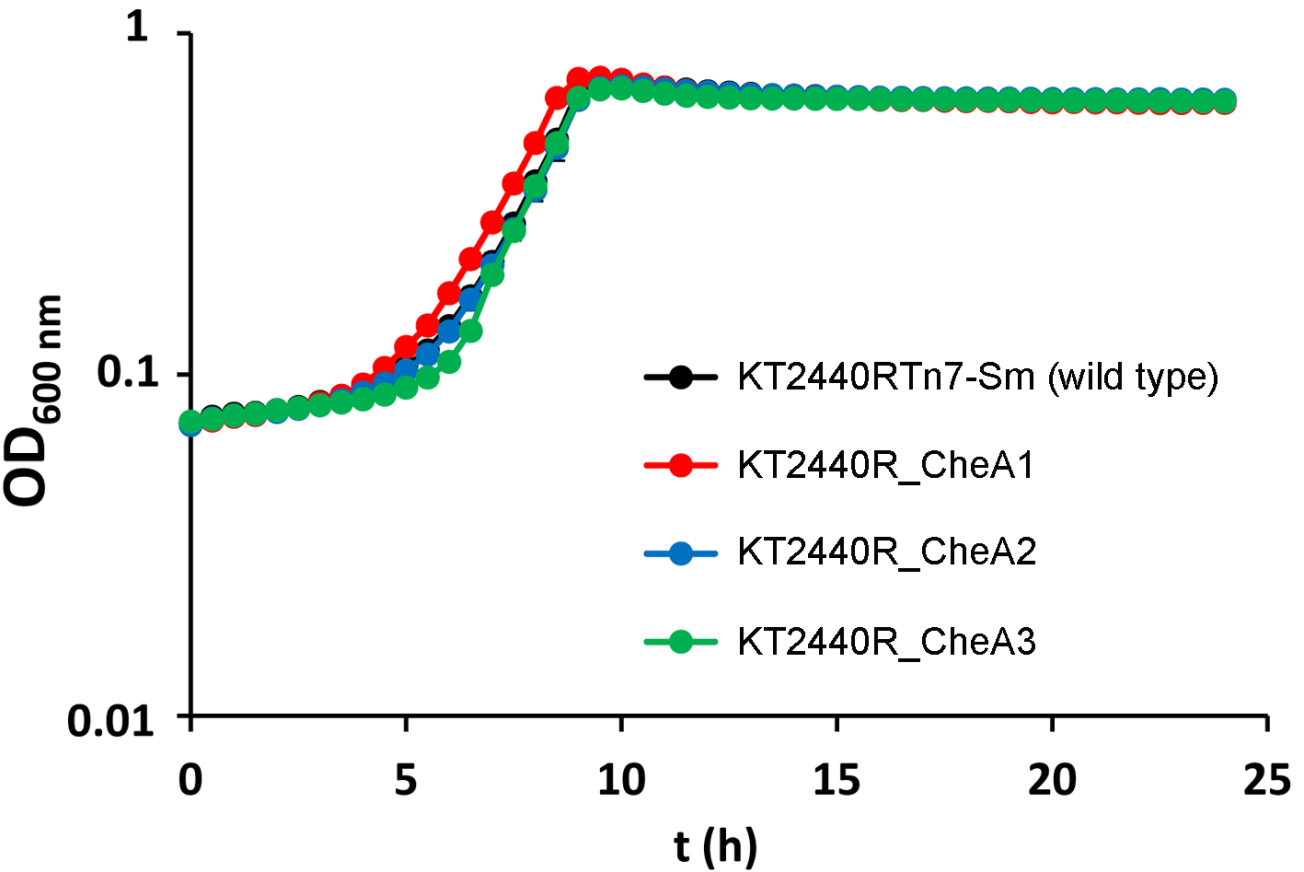
**

**Supp. Fig. 4) Growth kinetics of *Pseudomonas putida* KT2440RTn*7*-Sm (wild type) and derivative strains with mutations in *cheA* genes.** Bacterial strains were cultured at 30 ºC in minimal medium with 10 mM glucose as carbon source and growth was monitored using Bioscreen Microbiological Growth Analyser (Oy Growth Curves Ab Ltd, Helsinki, Finland). Shown are means and standard deviations of five biological replicates.

**Supp. Fig. 5)** **Analysis of the inocula used for the competitive root colonization assays.** The mixture of *P. putida* KT2440RTn*7*-Sm and *P. putida* mutants in the *cheA* genes were quantified by plating onto streptomycin and kanamycin containing agar plates. Supp. Fig. 4 illustrates that these strains show similar growth kinetics.

**Supp. Fig. 6) Halo diameters from swimming motility assays of *Pseudomonas putida* KT2440R and derivative *cheA* mutant strains.** Data are means and standard deviations of three biological replicates. Representative images of these assays are shown in Fig. 5C.

**Supp. Table 1) The relative abundance of compounds present in MRE.** Data were obtained by GC-MS analysis (Supp. Fig. 1). The relative abundance was estimated from the corresponding peak areas.

| Compound name | Relative abundance |
| --- | --- |
| **Sugars** |  |
| glucose | ++++ |
| maltose | +++ |
| galactose | +++ |
| fructose | +++ |
| ribose | ++ |
|  |  |
| **Sugar acids** |  |
| galactaric acid | ++ |
| threonic acid | ++ |
| gluconic acid | ++ |
|  |  |
| **Amino acids** |  |
| threonine | ++ |
| aspartic acid | ++ |
| glycine | + |
| glutamic acid | + |
| serine | + |
| glutamine | + |
| GABA | + |
|  |  |
| **Organic acids** |  |
| 2-keto-gluconic acid (isomer 1) | ++ |
| 2-keto-gluconic acid (isomer 2) | ++ |
| citric acid | ++ |
| lactic acid | ++ |
| succinic acid | ++ |
| methylmalonic acid | + |
|  |  |
| **Others** |  |
| glycerol | +++ |
| myo-inositol | ++ |
| urea | + |
